# Five novel mutations in the *ADAR1* gene associated with dyschromatosis symmetrica hereditaria

**DOI:** 10.1186/1471-2350-15-69

**Published:** 2014-06-20

**Authors:** Qi Liu, Zhen Wang, Yuhong Wu, Lihua Cao, Qingzhu Tang, Xuesha Xing, Hongwei Ma, Shifa Zhang, Yang Luo

**Affiliations:** 1 The Research Center for Medical Genomics, MOH Key Laboratory of Cell Biology and Key Laboratory of Medical Cell Biology, Ministry of Education, China Medical University, Shenyang 110001, China; 2 Department of Dermatology, Seventh People’s Hospital of Shenyang, Shenyang 110003, China; 3 Department of Developing Pediatrics, Shengjing Hospital, China Medical University, Shenyang 110004, China; 4 Department of Dermatology, Shenyang Army General Hospital, Shenyang 110045, China

**Keywords:** Dyschromatosis symmetrica hereditaria, *ADAR1*, *in vivo* mRNA assay, Exonic splicing mutation, *in vitro* expression assay

## Abstract

**Background:**

Dyschromatosis symmetrica hereditaria (DSH) is an autosomal dominantly inherited skin disease associated with mutations of *ADAR1*, the gene that encodes a double-stranded RNA-specific adenosine deaminase. The purpose of this study was to investigate the potential mutations in *ADAR1* in seven Chinese families with DSH.

**Methods:**

All the coding exons including adjacent intronic as well as 5′ and 3′ untranslated region (UTR) of *ADAR1* were screened by direct sequencing. Moreover, quantitative reverse-transcription polymerase chain (qRT-PCR) and Western blot were applied to determine the pathogenic effects associated with the mutations.

**Results:**

Molecular genetic investigations detected five novel mutations (c.556C > T, c.3001C > T, c.1936_1937insTG, c.1065_1068delGACA and c.1601G > A resulting in p.Gln186X, p.Arg1001Cys, p.Phe646LeufsX16, p.Asp357ArgfsX47 and p.Gly471AspfsX30 protein changes, respectively) as well as two previously reported (c.2744C > T and c.3463C > T causing p.Ser915Phe and p.Arg1155Trp protein changes, respectively). Among them, we found that the substitution c.1601G > A at the last nucleotide of exon 2 compromised the recognition of the splice donor site of intron 2, inducing an aberrant transcript with 190-bp deletion in exon 2 and causing an approximately 50% reduction of *ADAR1* mRNA level in affected individual. In addition, consistent with the predicted results, the expression patterns of other novel mutations were detected by Western blot.

**Conclusion:**

We identified five novel and two recurrent mutations of the *ADAR1* gene in seven Chinese families with DSH and investigated potential effects of the novel mutations in this study. Our study expands the database on mutations of *ADAR1* and for the first time, demonstrates the importance of exonic nucleotides at exon-intron junctions for *ADAR1* splicing.

## Background

Dyschromatosis symmetrica hereditaria (DSH, MIM #127400), also called symmetric dyschromatosis of the extremities, is characterized by hyperpigmented and hypopigmented macules on the face and dorsal aspects of the extremities. The skin lesions usually appear from infancy or early childhood, commonly stop spreading before adolescence and last for life [1]. Generally showing an autosomal dominant pattern of inheritance with high penetrance, DSH has been reported in various parts of the world, but predominantly in Japan and China [2,3]. RNA-specific adenosine deaminase 1 (ADAR1) gene, located on chromosome 1q21.3, was identified to be responsible for DSH [2].

*ADAR1*, also called *DSRAD* (double-stranded RNA-specific adenosine deaminase), spans 30 kb and contains 15 exons [4]. It encodes RNA-specific adenosine deaminase 1 composed of 1226 amino acid residues, with a calculated molecular mass of 139 kDa. Mammalian *ADAR1* shows ubiquitous expression and is supposed to be one of the housekeeping genes. The enzyme catalyses the deamination of adenosine (A) to isosine (I) in double-stranded RNA substrates, which results in the creation of alternative splicing sites or alternations of codon and thus lead to functional changes in proteins [4].

In this study, we investigated seven families with DSH in Chinese Han population and found five novel and two recurrent mutations. We applied *in vivo* mRNA assays and *in vitro* expression assays to investigate the effects of these novel mutations and discussed their potential pathogenic mechanisms.

## Methods

### Sample collection and ethics statement

**Figure 1:**
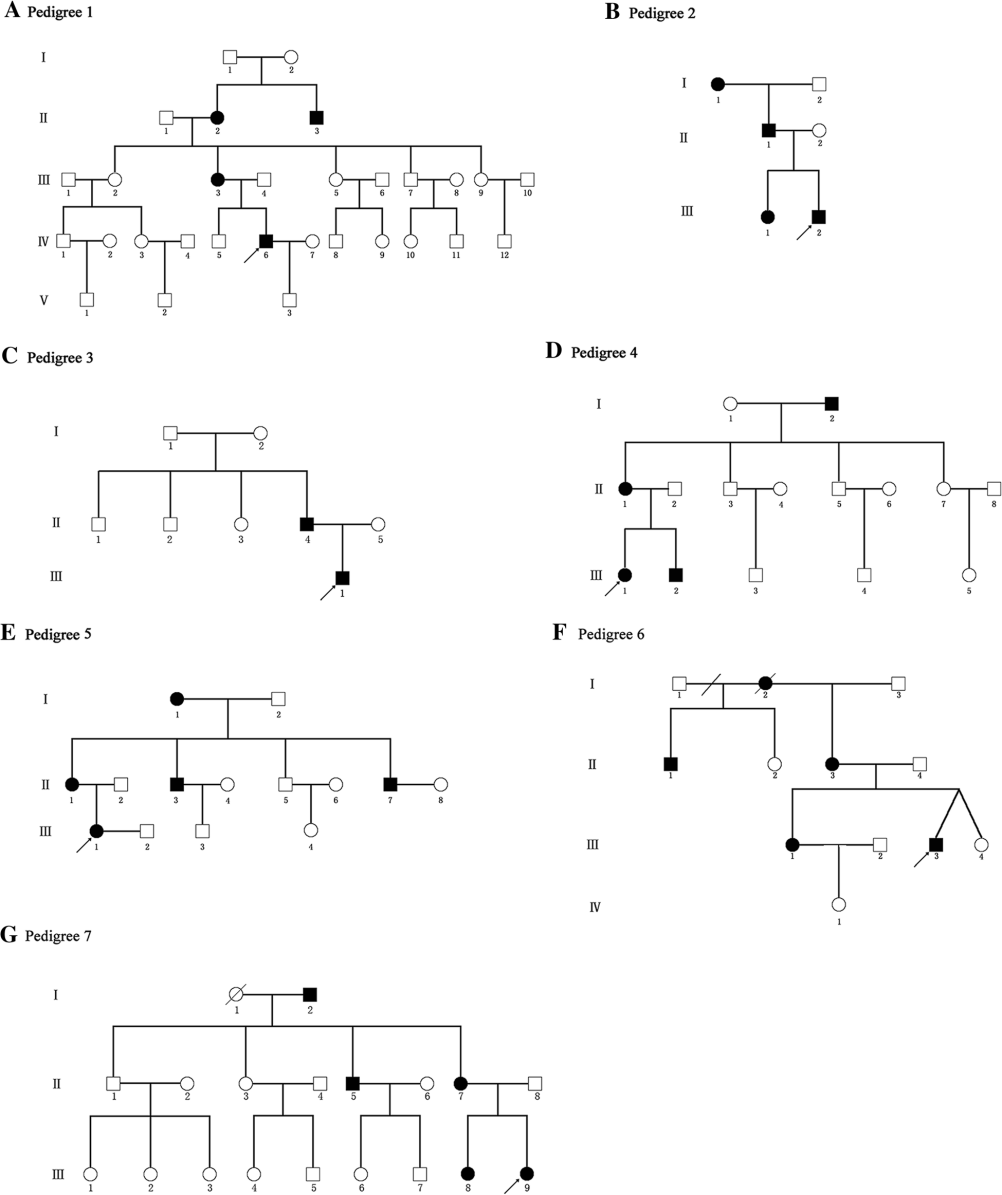
**Family Pedigrees in this study.** Seven multi-generation families with DSH illustrated autosomal dominant inherence pattern with 29 affected individuals. **(A-G)** Pedigrees of the studied DSH family 1-7. Affected family members are presented by black symbols. The probands are arrowed.

**Figure 2:**
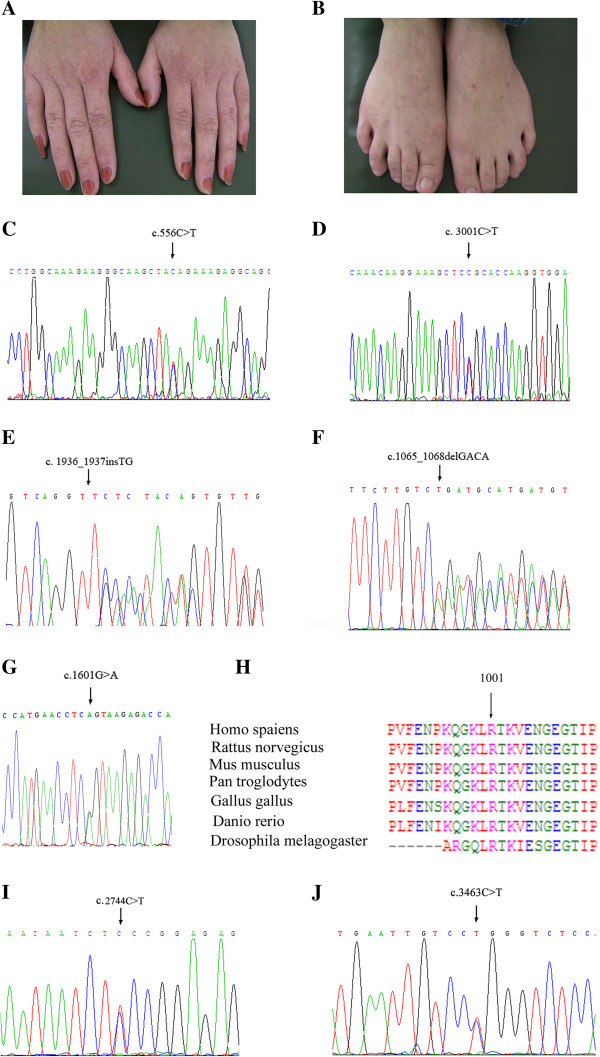
**Typical skin lesions and mutations of the*ADAR1*gene. (A, B)** A mixture of hyperpigmented and hypopigmented macules distributed on the dorsal aspects of hands and feet of the proband of family 4 (III1); **(C-G, I-J)** Direct DNA sequencing showing the five novel mutations in the family 1–5 **(C-G)** and the two recurrent mutations in family 6 and 7 **(I, J)**, respectively; **(H)** ClustaIW multiple alignment of various species around residue 1001 of human ADAR1.

Seven unrelated multi-generation DSH families exhibited an autosomal dominant inherence patterns (Figure 1). All affected individuals had typical macules on the dorsal aspects of the hands and feet. These lesions, with irregular shapes and sizes, appeared from early childhood, generally ranging from 3 to 10 years (Figure 2A, 2B). The skin lesions became more pronounced after sun exposure. No patient had extracutaneous symptoms. In family 3, the affected individuals had hypopigmented and hyperpigmented macules around ankle and in family 4 and 5, small freckle-like pigmented macules disturbed on the faces of affected individuals. DSH was diagnosed by experienced dermatologists based on the typical manifestations. The study was approved by the Research Ethics Committee of China Medical University and all the participants gave written informed consent.

### Extraction of genomic DNA and PCR

Genomic DNA samples were extracted from peripheral blood using the Universal Genomic DNA Extraction Kit (TaKaRa, Dalian, China). The primers that flanked all 15 coding exons and intron-exon boundaries of the *ADAR1* were designed (Table 1). Using Taq DNA polymerase, all fragments were amplified as follows: 1 min at 94°C, 30 cycles (30 sec at 94°C, 30 sec at 58°C, 1 min at 72°C) and 5 min at 72°C. After the amplification, the products were purified on agarose gels and subjected to sequencing.

**Table 1:** **Primers and conditions used in PCR for *ADAR1 *** **gene**

**Exon**	**Primers (5′ → 3′)**	**Product size (bp)**	**Annealing temperature (°C)**
1	Forward: ACTTCCAGTGCGGAGTAGCGGAG	221	66
	Reverse: CAAGACGCACACGCTACGCACTGC		
2	Forward: TCAAGGGCTGTTCCACAGGCAGC	1754	61
	Reverse: TCAGCCAAGACTGCGTCAGGAGC		
3-4	Forward: GGCAGAAGGAGTGACCTAGACTC	907	66
	Reverse: GGTCAATCTGCCATCCCTGAGGAGG		
5-6	Forward: AGAGGCTAGGTCAGGCTCCTCAGTC	674	66
	Reverse: TAAGAGGCCGTGGAGACAGGGCT		
7-8	Forward: CATATGTTTGCAAGACTGGCC	835	63
	Reverse: TGCATGGACTCCAGGGGAGGATGAG		
9	Forward: CTGAGGCTGTTTCTGCCTTGAAGC	253	63
	Reverse: GGGAACTGGAGCTCTCCACAG		
10	Forward: GCCCTCAGCAGAAATGAAGGAGACCC	456	65
	Reverse: CTCAAACCCACAGTGGAGTGTGGC		
11	Forward: CTGCTGTCCACCTCCAGTCTCCCAT	385	66
	Reverse: CAGCACCTCTGTGCCCAGTGAC		
12	Forward: GTGGTTTGGTCCATTGGCGCCTGTG	300	66
	Reverse: GCATGCAGTTTGGGATCTGGGCAC		
13-14	Forward: CTGGTAGCCCCAGTCAGAAGGTGCT	864	66
	Reverse: GATGCACCCTTGCAAGTCAGGGCAG		
15	Forward: ACGGTGTCTCCACTGTGAGCTCCT	372	63
	Reverse: GCTACGACCTACCTCTCTCACACCC		

### RNA splicing analysis

Fresh peripheral blood samples were collected from the affected individual and 3 healthy controls. Total RNA was extracted using the TRIzol reagent according to the manufacturer’s protocols. After quantification, 1 μg of RNA sample was reversely transcribed into cDNA using an AMV reverse transcriptase kit from TaKaRa Company according to the manufacturer’s instructions. To analyze the expression of the mutant *ADAR1* allele, RT-PCR was performed with primers E2F/E3R (Table 2) [5]. The cDNA fragments were then subcloned into pMD 18-T vector and subjected with sequencing. Moreover, RT-PCR was carried out with primer pairs E1F/E3R and E1F/E4R to determine whether there was exon skipping in affected individual.

**Table 2:** Primer sequences used in RT-PCR and real-time quantitative RT-PCR

RT-PCR	E1F: 5′-GAGGAAACGAAAGCGAAATTGAACCGGA-3′
	E2F: 5′- AACTCCACATCTGCCTTGGAAGATCCTC -3′
	E3R: 5′-CTCTCGGCCATTGATGACAACCTGGAAT-3′
	E4R: 5′-TGTGCATACACTCAAGCAGTGTGGTGAC-3′
Real-time RT-PCR	E9-10 F: 5′-GGCTTCATCAGGTTTCTCTAC-3′
	E10-11R: 5′-CACGGAGCAGTGCTGATATAC-3′
	GAPDH-F: 5′-CATCTTCCAGGAGCGAGATC-3′
	GAPDH-R: 5′-GCAAATGAGCCCCAGCCTTC-3′

### Real-time quantitative RT-PCR

To quantify the relative mRNA expression of *ADAR1* in affected individual, real-time quantitative RT-PCR was carried out using primer pairs E9-10 F/E10-11R (Table 2). The amplicon of *ADAR1* was 140 bp in length, normalized by a 120-bp *glyceraldehyde-3-phosphate dehydrogenase* (*GAPDH*) fragment. Reactions were performed with SYBR Green PCR Master Mix (TaKaRa) [5]. Three replicates of each reaction were carried out and the 2^-ΔΔCT^ method was used to calculate relative changes in *ADAR1* expression. Melting curve analysis confirmed that only the expected product was amplified [5].

### *In vitro* expression assay

The wild-type coding sequence of *ADAR1* was amplified from pCMV6-ADAR1 (Origene) by PCR and subcloned into pFLAG-CMV-2 (Sigma-Aldrich, St. Louis, MO, USA) at its *Not* I restriction site by standard procedure. To generate mutant plasmids, site-directed mutagenesis using PCR-based method was applied. Concisely, primers were designed for mutagenesis and fragments amplified by PCR were subcloned into pFLAG-CMV-2 vector using a standard subcloning protocol, respectively. After confirmed by sequencing, all plasmids were transfected into human embryonic kidney (HEK-293) cells, respectively [6]. The whole cellular lysates were extracted at 48 h post-transfection and immunoblotting was performed as reported previously [7].

## Results

### Mutation analysis

Among 7 Chinese families with DSH, we found 5 novel mutations in *ADAR1* (Table 3). The spectrum of mutations included one nonsense mutation, two missense mutations, one small insertion and one small deletion, all of which were heterozygous (Figure 2C-G). Searching the Human Gene Mutation Database (HGMD) and PubMed, we did not find the reports about these variants. All the mutations segregated affected individuals from 100 normal Chinese subjects, suggesting that neither of them was a polymorphism (For point mutations c.556C > T, c.3001C > T and c. 1601G > A, PCR-RFLP was applied with restriction endonuclease *Bsp*1407I, *Bgl* II and *Sca* I while for small insertion/deletion c.1936_1937insTG and c.1065_1068delGACA, SDS-PAGE was applied after PCR amplification, respectively).

**Table 3:** **Mutations of the *ADAR1 *** **gene identified in this study**

**Patient**	**Part of lesions**	**Mutation**			**Mutation effect**
		**Nucleotide changes**	**Amino-acid changes**	**Exon**	
1	Back of hands and feet	c.556C > T	p.Gln186X	2	Nonsense
2	Back of hands and feet	c.3001C > T	p.Arg1001Cys	11	Missense
3	Back of hands and feet, ankles	c.1936_1937insTG	p.Phe646LeufsX16	5	Frameshift
4	Back of hands and feet, face	c.1065_1068delGACA	p.Asp357ArgfsX47	2	Frameshift
5	Back of hands and feet, face	c.1601G > A	p.Gly471AspfsX30	2	Splicing
6	Back of hands and feet	c.2744C > T	p.Ser915Phe	9	Missense
7	Back of hands and feet	c.3463C > T	p.Arg1155Trp	15	Missense

In the family 1, the nonsense mutation was detected in exon 2 of *ADAR1* and defined as c.556C > T (Figure 2C). This mutation was suggested leading to a truncated ADAR1 protein lacking 1040 amino acid residues. In the family 2, the missense mutation in exon 11, c.3001C > T, was identified by sequencing (Figure 2D). This mutation replaced arginine residue with cysteine, which was shown as a highly conserved residue within the functional domain of the ADAR1 (Figure 2H).Moreover, nucleotides TG were found to insert between c.1936 and c.1937 in the family 3 and this 2-bp small insertion induced a frameshift from codon 646 and expected to produce a premature termination codon (PTC) at codon 661 (Figure 2E). In addition, one 4-bp small deletion, c.1065_1068delGACA, was detected in the family 4. This mutation might shift the reading frame and generate a PTC at codon 403 (Figure 2F).

Furthermore, direct sequencing of *ADAR1* revealed a missense substitution, c. 1601G > A, at the end of exon 2 in the family 5 (Figure 2G). Since this G nucleotide belongs to the conserved exon-intron junction, we performed splicing predictions with web-based resources. Analysis with SpliceView (http://zeus2.itb.cnr.it/~webgene/wwwspliceview_ex.html) and Human Splicing Finder (http://www.umd.be/HSF/) indicated that the recognition of such splice donor site was potentially attenuated in the presence of c.1601G > A, supporting that this mutation might play a role in splicing process.

Moreover, two recurrent mutations, c.2744C > T and c.3463C > T, were detected in family 6 and 7, respectively (Figure 2I, 2J). Both of them were missense mutations, suggesting the replacements of serine 915 by phenylalanine and arginine 1155 by tryptophan, respectively [8,9].

### Molecular analysis of expression patterns of the novel mutations

**Figure 3:**
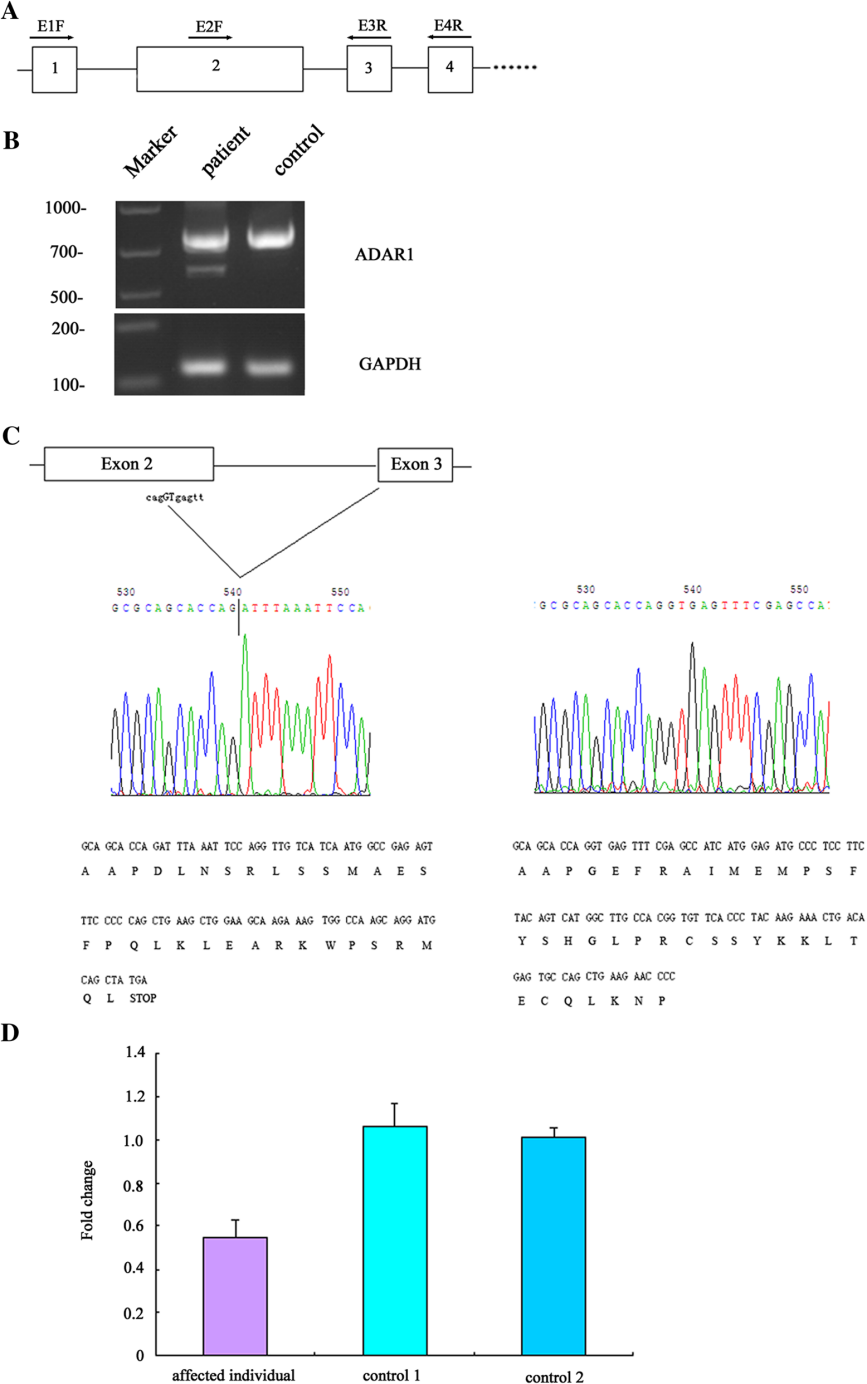
**Aberrant splicing pattern of *ADAR1 *caused by c.1601G > A mutation. (A)** Locations of primers used in RT-PCR; **(B)** RT-PCR analysis revealed a transcript abnormality in *ADAR1*; **(C)** cDNA sequencing and predicted amino acid sequence of the aberrant (left) and normal (right) transcripts of *ADAR1*; **(D)** The relative quantities of *ADAR1* mRNA level derived from real-time quantitative RT-PCR.

Furthermore, we attempted to determine the exact consequence of the c.1601G > A mutation in splicing with *in vivo* mRNA assays. RT-PCR analysis revealed differential splicing patterns between affected individual and healthy control. While predicted 780-bp PCR fragments of normal splicing were shown and confirmed by sequencing analysis both in the affected and healthy individual, an additional about 600-bp PCR product was shown in the presence of mutant and subsequently subcloned into pMD18-T (Figure 3A, 3B). Direct sequencing of TA clones revealed that this mutation caused a splicing error in *ADAR1*. With functional loss of the native intron 2 donor site, this mutation activated the cryptic splicing donor site GT lying upstream of the normal splicing donor site in exon 2 and thus caused a 190-bp deletion in mRNA sequence (Figure 3C). Hence, this c.1601G > A mutation was expected to shift the reading frame from codon 471 with 29 aberrant amino acids and generate premature termination at codon 500. Moreover, no additional band was detected in RT-PCR assays using the primer pairs E1F/E3R and E1F/E4R, excluding the possibility of exon skipping in affected individual due to this mutation (data not shown).

Upon the worthwhile observation that the aberrant transcript was present at much reduced level in the affected individual, mRNA quantification was evaluated with real-time quantitative RT-PCR. By validating with the 2^-ΔΔCT^ method, the relative quantities were 0.525, 0.639 and 0.467 (mean ± SD, 0.54 ± 0.09) for individual carrying with c.1601G > A mutation compared with healthy subject, indicating an approximately 50% reduction of the *ADAR1* mRNA expression in affected individual.

**Figure 4:**
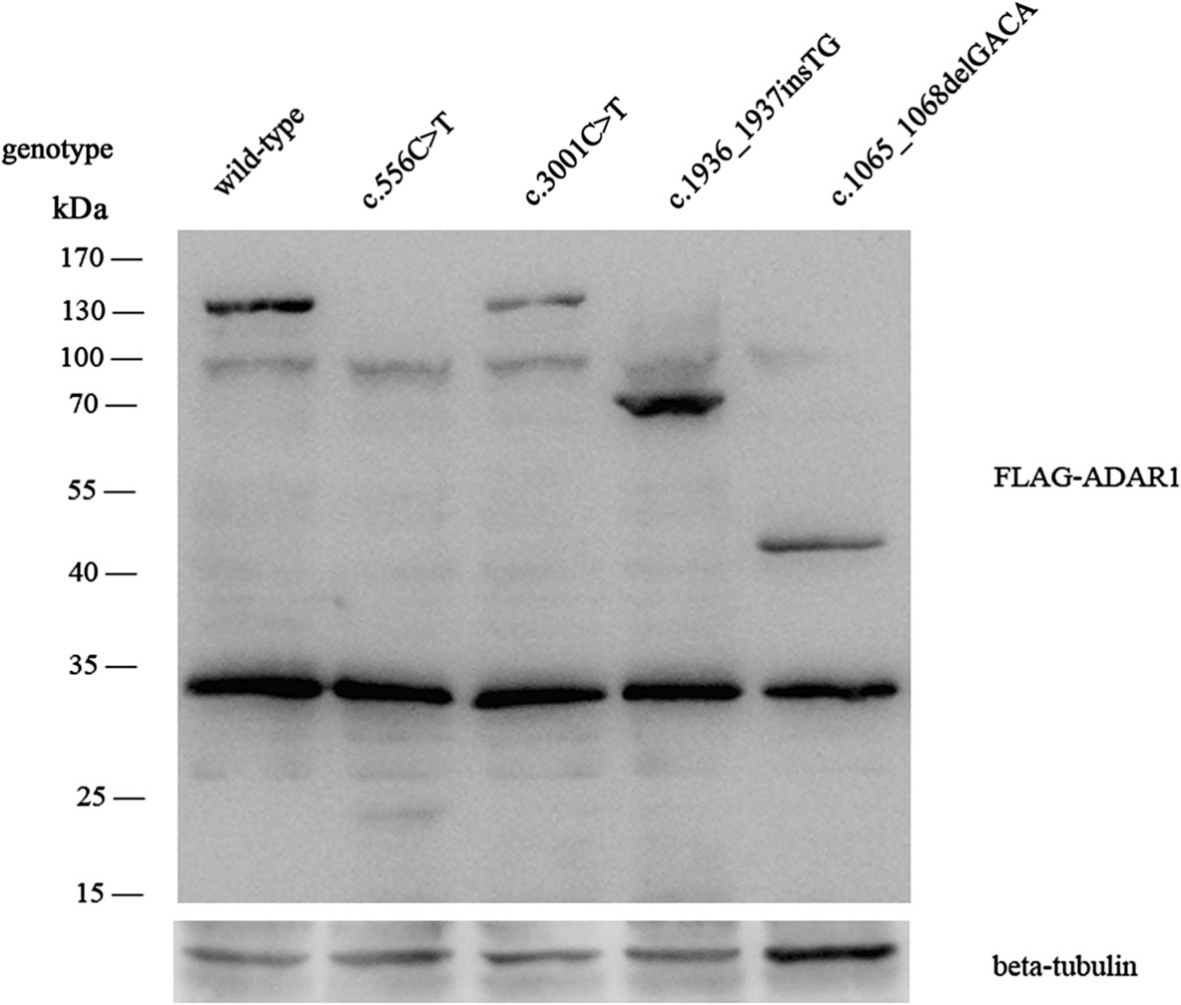
***In vitro *expression assays.** Total cellular lysates of HEK-293 cells transfected with FLAG-tagged wild-type or mutant plasmids were immunoblotted with FLAG antibody. beta-tubulin was used as an internal control.

Since fresh blood samples from affected individuals of family 1 to 4 were not available, we continued to investigate the expression patterns of these mutants with *in vitro* expression assays. Briefly, immunoblottings were performed with lysates of HEK-293 cells overexpressing wild-type or mutants of *ADAR1*. Consistent with the predicted results, the truncated ADAR1 proteins were detected as 21 kDa in cells overexpressing c.556C > T mutation, 75 kDa in cells overexpressing c.1936_1937insTG mutation and 46 kDa in cells overexpressing c.1065_1068delGACA mutation, respectively (Figure 4). Moreover, as a missense mutation, c.3001C > T (p.Arg1001Cys) mutant demonstrated the same molecular weight as the wild-type ADAR1 protein.

## Discussion

A-to-I RNA editing is one of the post-transcriptional modifications widespread in mammals, which modifies adenosine (A) base in pre-mRNA to yield inosine (I) [10]. Since I is recognized as guanosine during translation, A-to-I RNA editing in coding sequences probably leads to amino acid changes which often entails changes in protein function [10]. Moreover, editing A-to-I modification might alter splice patterns as well as RNA structures and stabilities [1,10,11]. A-to-I editing is catalyzed by ADARs in vertebrate and till now, three ADAR family members, ADAR 1–3, have been identified. Among them, *ADAR1* was confirmed to be responsible for DSH in 2003 [1]. To date, the mechanisms by which *ADAR1* mutations cause DSH still remain unclear [10,11].

Human ADAR1 contains, from N- to C-terminals, two adenosine deaminase Z-alpha (Zα) domains, three double-stranded RNA binding motifs (DSRMs) and a tRNA-specific and double-stranded RNA adenosine deaminase (ADEAMc) domain, which encodes by exon 2, 2–7 and 9–15 of *ADAR1*, respectively [5]. Although ADEAMc is the catalyze domain for A-to-I modification, Zα and DSRM domains are also essential for the activity of ADAR1 [5,11-14].

**Figure 5:**
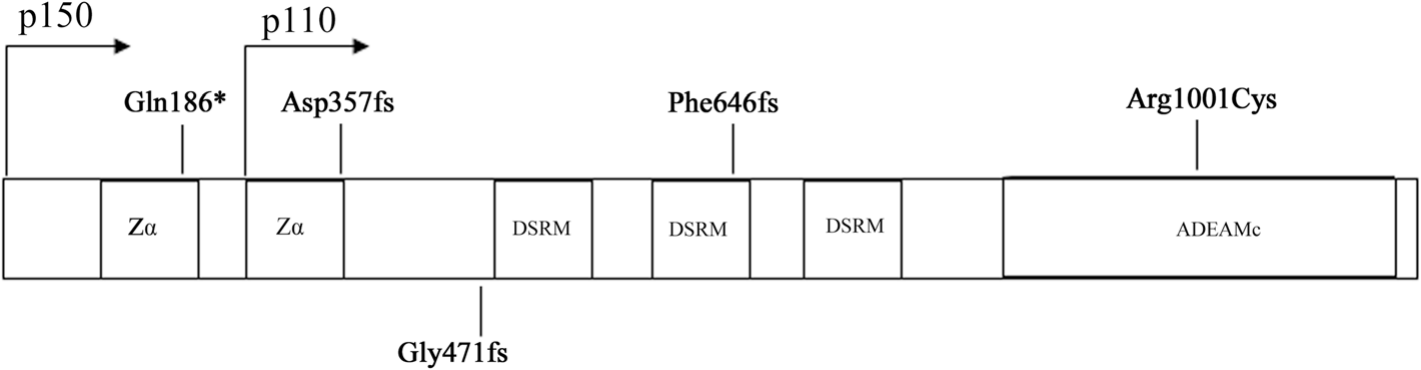
**Novel mutations of *ADAR1 *identified in this study.** The functional domains (Zα, DSRM, ADEAMc) were indicated in the figure. Zα: Z-DNA binding domains; DSRM: double stranded RNA binding domains; ADEAMc: deaminase domain.

In this study, we reported five novel mutations and two recurrent mutations. Among them, the nonsense mutation c.556C > T (p.Gln186X) and small deletion c.1065_1068delGACA (p.Asp357ArgfsX47) are expected to cause ADAR1 truncations lacking the entire three DSRMs and ADEAMc domain while c.1936_1937insTG (p.Phe646LeufsX16), the small insertion identified in our study, supposes to change the reading frame from the second DSRM and induces truncated ADAR1 protein without the last DSRM and ADEAMc domain (Figure 5). In addition, Figure 4 suggested that the mutations c.556C > T and c.1065_1068delGACA might reduce the expression of mutant proteins and we speculated that the nonsense-mediated mRNA decay (NMD) might be one of the possibilities. For the missense mutations c.3001C > T (p.Arg1001Cys), c.2744C > T (p.Ser915Phe) and c.3463C > T (p.Arg1155Trp), the transitions of nucleotides change codons in the highly conserved region of ADEAMc domain, which possibly alter the activity of ADAR1 directly, or by disturbing the formation of wild-type ADAR1 homodimers [9]. In family 5, the aberrant splicing decreased approximately half *ADAR1* mRNA level, potentially caused by NMD, supporting that haploinsufficiency of *ADAR1* produces DSH [5,9]. In addition, we compared the clinical features with the mutations indentified in all the families but failed to find any clear relationship between phenotypes and genotypes as other groups have reported [2,13].

Till now, about 145 *ADAR1* mutations have been reported in DSH patients, which consist of 80 missense and nonsense mutations, 11 splicing mutations, 37 small deletions, 14 small insertions, 2 small indels and 1 gross insertion [15-29]. So far, most known missense mutations (49 in 57, 86%) are located within the ADEAMc domain encompassing amino acids 839–1222 [3]. The missense mutations identified in our study are also located in this domain. These data support that the ADEAMc domain is critical for this enzyme and indicates that this region might be hot spot for mutation within *ADAR1*[10,16,17]. To date, 23 nonsense mutations have been reported in *ADAR1* and they appear to distribute throughout the gene without apparent unifying connection. Till now, 11 splicing mutations have been identified, all of which situate in *ADAR1* introns. Among them, only 4 mutations were further studied with cDNA sequence analysis and detected the aberrant splicing products [20,21,27], in which 3 splicing mutations led to skipping one or more exons near to the mutations [27], and the other one was a in-frame mutation which generated a 4-amino acid deletion in ADAR1 protein [21]. To our knowledge, the c.1601G > A mutation identified in our study is the first report about the exonic splicing mutation in *ADAR1*.

Although ADAR1 expressed ubiquitously in mammalian tissues and playing various roles such as viral host defense, embryonic development, tumor growth and miRNA processing [22-25], only a few its target genes including the neurotransmitter receptors in the central nervous system and the hepatitis virus antigen in the liver are identified [9]. Therefore, mutations in the *ADAR1* might associate with neurological dysfunction such as dystonia and mental deterioration [26]. However, like most affected individuals reported previously, patients in our study only show pigmentary abnormality in skin without obvious dysfunction in extracutaneous tissues. Recently, mutations identified in *ADAR1* were reported to be relevant with autoimmune disorder, hair color change and dental abnormality [15,27]. Thus, further investigation on the mechanisms by which ADAR1 regulates its target genes in different tissues will facilitate the understanding of ADAR1 functions.

Driven by different promoter, ADAR1 has been characterized into two isoforms, an interferon-inducible 150 kDa protein (p150) and a constitutively-expressed 110 kDa protein (p110), in mammalian cells [1,2]. The p150 isoform, translated from the methionine initiation codon, is the full length protein mainly distributed in cytosol while the p110 isoform initially translates from the AUG at codon 296 and locates in the nucleus [28]. Till now, including p.Gln186X identified in our study, four mutations of *ADAR1* have been reported at the 5′ side of codon 296 [2,29]. However, the distinct roles of the isoforms are obscure. Recently, Zhang et al. reported that the small deletion p.R91fsX123 could eliminate the expression of the p150 transcript through NMD, while did not significantly alter the expression of p110 in patients of DSH, indicating that p150 isoform of ADAR1 might be the determinant of DSH [29].

## Conclusion

In conclusion, we identified five novel and two recurrent mutations of the *ADAR1* gene in seven Chinese families with DSH and investigated potential effects of the novel mutations in this study. Together with previous related studies, these results might give insight into the still unknown mechanisms leading to DSH. Additionally, our results enrich the *ADAR1* mutation database and will contribute further to the understanding of DSH genotype/phenotype correlations as well as to the pathogenesis of this disease.

## Abbreviations

DSH: Dyschromatosis symmetrica herediaria; ADAR1: RNA-specific adenosine deaminase 1; PTC: Premature termination codon; NMD: Nonsense-mediated mRNA decay.

## Competing interests

The authors declare that they have no competing interests.

## Authors’ contributions

QL participated in the design of this study, performed the statistical analysis and drafted the manuscript. QL, YHW and LHC carried out the molecular genetic study. XSX and QZT performed sequence alignment. ZW, HWM and SFZ diagnosed the patients and collected clinical data. YL conceived of the study, participated in the design and coordination and helped the draft the manuscript. All authors read and approved the final manuscript.

## Pre-publication history

The pre-publication history for this paper can be accessed here:

http://www.biomedcentral.com/1471-2350/15/69/prepub
